# Discovering Tuberosin and Villosol as Potent and Selective Inhibitors of AKT1 for Therapeutic Targeting of Oral Squamous Cell Carcinoma

**DOI:** 10.3390/jpm12071083

**Published:** 2022-06-30

**Authors:** Mohd Adnan, Deeba Shamim Jairajpuri, Muskan Chaddha, Mohd Shahnawaz Khan, Dharmendra Kumar Yadav, Taj Mohammad, Abdelbaset Mohamed Elasbali, Waleed Abu Al-Soud, Salem Hussain Alharethi, Md. Imtaiyaz Hassan

**Affiliations:** 1 Department of Biology, College of Science, University of Hail, Hail P.O. Box 2440, Saudi Arabia; drmohdadnan@gmail.com; 2 Department of Medical Biochemistry, College of Medicine and Medical Sciences, Arabian Gulf University, Manama 26671, Bahrain; deebasj@agu.edu.bh; 3 Centre for Interdisciplinary Research in Basic Sciences, Jamia Millia Islamia, Jamia Nagar, New Delhi 110025, India; muskan.chaddha.mc@gmail.com (M.C.); taj144796@st.jmi.ac.in (T.M.); 4 Department of Biochemistry, College of Science, King Saud University, Riyadh 11451, Saudi Arabia; moskhan@ksu.edu.sa; 5 College of Pharmacy, Gachon University of Medicine and Science, Hambakmoeiro, Yeonsu-gu, Incheon 21924, Korea; 6 Department of Clinical Laboratory Science, College of Applied Sciences-Qurayyat, Jouf University, Sakaka 72388, Saudi Arabia; aeelasbali@ju.edu.sa; 7 Department of Clinical Laboratory Sciences, Faculty of Applied Medical Sciences, Jouf University, Sakaka 72388, Saudi Arabia; wabualsoud@ju.edu.sa; 8 Health Sciences Research Unit, Jouf University, Sakaka 72388, Saudi Arabia; 9 Department of Biological Science, College of Arts and Science, Najran University, Najran 66252, Saudi Arabia; shalharthi@nu.edu.sa

**Keywords:** AKT1, oral squamous cell carcinoma, personalized medicine, natural compounds, drug discovery, virtual screening, molecular dynamics simulation

## Abstract

Oral squamous cell carcinoma (OSCC) is a major cause of death in developing countries because of high tobacco consumption. RAC-alpha serine-threonine kinase (AKT1) is considered as an attractive drug target because its prolonged activation and overexpression are associated with cancer progression and metastasis. In addition, several AKT1 inhibitors are being developed to control OSCC and other associated forms of cancers. We performed a screening of the IMPPAT (Indian Medicinal Plants, Phytochemistry and Therapeutics) database to discover promising AKT1 inhibitors which pass through various important filters such as ADMET (absorption, distribution, metabolism, excretion, and toxicity) properties, physicochemical properties, PAINS (pan-assay interference compounds) filters, PASS (prediction of activity spectra for substances) analysis, and specific interactions with AKT1. Molecules bearing admirable binding affinity and specificity towards AKT1 were selected for further analysis. Initially, we identified 30 natural compounds bearing appreciable affinity and specific interaction with AKT1. Finally, tuberosin and villosol were selected as potent and selective AKT1 inhibitors. To obtain deeper insights into binding mechanism and selectivity, we performed an all-atom molecular dynamics (MD) simulation and principal component analysis (PCA). We observed that both tuberosin and villosol strongly bind to AKT1, and their complexes were stable throughout the simulation trajectories. Our in-depth structure analysis suggested that tuberosin and villosol could be further exploited in the therapeutic targeting of OSCC and other cancers after further clinical validations.

## 1. Introduction

Cancer is a conglomeration of diseases that develop over a period of time and progress into a state of uncontrolled division of cells [1]. Such cells are functionally impaired and therefore cannot behave in the required manner. Such a state is harmful to physiological and molecular processes that sustain a living organism [2]. The occurrence of cancer is attributed to the alterations that take place in one or many processes that regulate cell division and cell death (apoptosis) [3]. Cancer requires constant communication between different factors and proteins that are involved in the regulation of tumor growth and vascular permeability that support its spread. Src family kinases (SFKs) control the vascular permeability of tumors along with tumor growth, metastasis, and endothelial-barrier regulation [4]. Similar to SFKs, protein kinase B also plays a crucial role in establishing communication between the factors promoting tumor growth and metastasis. Protein kinase B, or AKT, is a type of serine-threonine kinase [4,5].

The serine-threonine kinases have a key role in the management of processes such as programmed cell death, cell differentiation, cell proliferation, and embryonic development [6]. The protein kinase B exists in three isoforms that are AKT1, AKT2, and AKT3 [7]. Among them, AKT1 plays crucial functions in various activities related to the cell cycle, including, cell proliferation, development of blood vessels (angiogenesis), cell growth, and survival [8]. It also participates in maintaining glucose levels through insulin-induced translocation of the specific glucose transporter, SLC2A4/GLUT, to the surface of the cell [9]. The overexpression of AKT1 is linked to the development of oral squamous cell carcinoma (OSCC) [10,11] and many other complex diseases [12,13,14]. However, although it is involved in providing resistance against radiation, its inhibition is linked to the cessation of important pathways required for cell survival and overactivation of signal transduction pathways such as the receptor tyrosine kinase [15]. Studies show that reduced expression of AKT1 in knockdown mice also reduced the expression of TGFBR1 (transforming growth factor beta receptor 1) which is expressed in malignant phenotypes of OSCC [10]. 

The three AKT isoforms have also been studied and their role in the pathogenesis of cancer has been found through rigorous evaluation [16]. The intricate molecular network through which these isoforms work in triggering the oral squamous cell carcinoma can provide a means to effectively target and treat such challenging diseases [17]. The AKT1 protein isoforms are phosphorylated through the intervention of phosphoinositide 3-kinase, or PI3K [18]. The AKT1/PI3K signaling complex is involved in the regulation of processes such as angiogenesis [19]. Thus, this signaling complex forms a crucial part of various signaling cascades including G protein-coupled receptor (GPCR) signaling, receptor tyrosine pathway, and integrin-linked kinase [20]. AKT, therefore, has a crucial role in the induction of tumorigenesis by acting on different pathways that can lead to cancer development. Owing to its remarkable role in cancer progression, AKT1 represents itself as a potential druggable target in anticancer therapeutics [21,22]. 

High oxidative stress is linked to an increase in the rate of cell proliferation, metastasis, cell survival, and angiogenesis [23]. Cellular oxidative stress also hampers cell death signaling and increases resistance to biochemical drugs [24]. Therefore, reducing the reactive oxygen species and maintaining mitochondrial membrane potential can aid in decreased oxidative stress [25]. Phytoconstituents progressively emerged as safe and effective agents against anticancer drug development [26,27,28]. Tuberosin (5 hydroxy 3,4,7,3′,4′ pentamethoxy flavone) is extracted from the Indian medicinal plants *Calopogonium mucunoides* and *Pueraria tuberosa* [29]. It exhibits antioxidant properties, acts as a biochemical scavenger against hydroxyl radicals and superoxide radicals, and performs metal chelation [30]. Villosol, obtained from *Tephrosia purpurea*, possesses anti-inflammatory and anticancer properties and can serve as an important inhibitor of inflammation and oxidative damage that is linked to the development of cancer [31].

Although there are a few inhibitors already known for AKT1, still, new discoveries are required for the development of novel, safe, and effective drugs against AKT1-mediated malignancies [32]. Computational methods involving the use of virtual screening and molecular dynamics (MD) simulation are commendable for discovering potential inhibitors of predefined targets [33,34,35,36]. We performed a systematic structure-guided screening of natural compounds from the IMPPAT (Indian Medicinal Plants, Phytochemistry and Therapeutics) database to find out the most promising compounds that can help in the inhibition of AKT1, which is involved in the pathways leading to increased cell survival in cancer development.

## 2. Materials and Methods

### 2.1. Virtual Screening

The crystal structure of a protein is a prerequisite for molecular docking and, thus, the PDB file for the crystal structure of AKT1 was retrieved from the RCSB Protein Data Bank (PDB ID: 4GV1). The protein was assembled for virtual screening with the help of MGLTools [37], Swiss-PdbViewer [38], and InstaDock [39]. This step was crucial for filling gaps in the protein structure, adding hydrogen atoms to polar groups, and incorporating appropriate atoms. The molecular docking was conducted through InstaDock software under a blind search space. The visualization and interaction analysis was performed through DS Visualizer [40]. 

The phytoconstituents library was extracted from the IMPPAT database [41] after applying a filter following Lipinski’s rule of five [42]. The IMPPAT database contains a plethora of naturally derived plant compounds that possess pharmacological activity. The process of molecular docking was carried out to screen compounds that exhibit higher binding affinity towards the target protein AKT1. The compounds were filtered after using various segregating criteria, including binding affinity and ligand efficiency, and the most promising 30 candidates were taken ahead for further investigation. The compounds that exhibited acceptable affinity scores were processed and finalized to be investigated through InstaDock. 

### 2.2. PAINS Patterns and ADMET Properties of Compounds

Safety and efficacy are two very important pillars in drug discovery and development [43]. The compounds that are to be used for treating and curing any ailment should possess specificity and selectivity so that they only target the process or the protein they are designed to affect [44,45,46,47]. Multiple attacking sites can pose a threat to the various vital processes in the body and can do more damage than inferring the desired effect. The compounds selected from the molecular-docking-based screening of the IMPPAT database were further investigated to carefully analyze their physicochemical properties. Top hits were collected from InstaDock and were taken for PAINS analysis [48]. The PAINS filter helps in identifying pan-assay interference compounds which might have a higher tendency to bind with multiple targets. 

The compounds from the PAINS filter were further passed through an ADMET analysis which evaluates the absorption, digestion, metabolism, excretion, and toxicity of a chemical molecule. To carry out the ADMET analysis, the pkCSM [49] and SwissADME [50] servers were utilized. These software predict the pharmacokinetic and toxic properties of small molecules through graph-based signatures [51,52,53]. The software provides information on the efficacy of the compound through parameters such as total clearance, AMES toxicity, water solubility, skin permeability, hepatic toxicity, and so on. AMES toxicity helps to assess the probable carcinogenic effect of a compound through the use of the bacteria *S. typhi*. It is an effective and reliable method to estimate the genotoxicity of the compound and its propensity to reverse the mutations at the selected target in the bacterial strain [54,55,56]. We selected GI absorption, BBB permeation, CYP2D inhibitor, OCT2 substrate, AMES, and hepatotoxicity to finalize the compounds that showed the most promising results for effective molecules. The file format for pkCSM was selected as the SMILES format that was obtained from the Discovery Studio Visualizer software. 

### 2.3. Biological Activity Prediction through PASS

The PASS (prediction of activity of spectra for substance) analysis is crucial for identifying the biological properties of a chemical compound [57]. The PASS analysis carefully assesses the biological and pharmacological properties of compounds that are already selected after PAINS and ADMET screening. The results that were obtained from the PASS analysis were represented in the form of two parameters, “Pa” or probability of being active and “Pi” or probability of being inactive. The ratio between the two values was also displayed to indicate the biological potential of the molecule. The molecules with a higher Pa value indicated a greater and better biological property. 

### 2.4. Interaction Analysis

PyMOL was utilized for protein visualization and for exploring the possible interactions between the ligands and the shortlisted compounds. Apart from PyMOL, DS Visualizer served as an equally important tool for identifying probable interactions between the protein and ligands and in the identification of the important amino acid residues that participated in the formation of the binding pocket of the target protein. 

### 2.5. MD Simulations

The key to a successful understanding of the protein and ligand relationship is found through the all-atom simulation studies [34,36,58]. The GROMACS simulation suite [59] enabled performance force-field-aided simulation in the AKT1 kinase and its complexes with the selected ligands villosol and tuberosin. Out of all possible conformations from the docked compounds, the top conformation of each compound was selected for simulation as they exhibited the most promising interactive results with the active sites of the target protein, AKT1. The ligand complex topologies were generated through the PRODRG server [60]. For solvation, we placed each system in a cubic box at a distance of 10Å from the center to the edges while utilizing the simple point-charge (SPC216) water model. The steepest descent algorithm provided energy minimization and position refinement, followed by NVT and NPT ensembles. The MD run was performed for 100 ns and resultant trajectories were analyzed through the QtGrace software [61].

## 3. Results and Discussion 

### 3.1. Virtual Screening

The IMPPAT database served as a source of ~6000 compounds that conform to Lipinski’s rule of five (RO5) [41]. These compounds were selected after careful screening through advanced search filters such as Lipinski’s rule of five, the number of heavy atoms and heteroatoms, and stereochemical complexity. A molecular-docking-based virtual screening of filtered compounds resulted in the identification of 30 compounds selected based on their binding affinity scores (Table 1). High-affinity compounds might have a high tendency to bind in the active site pocket of AKT1 for its subsequent inhibition [55,56,62]. The docking study showed that all the selected hits exhibited appreciable binding affinity and ligand efficiency towards AKT1.

### 3.2. ADMET Properties and PAINS of the Compounds

The high-affinity compounds were subjected to the PAINS filter, where 16 out of 30 compounds were passed and further subjected to ADMET analysis. ADMET analysis aided in selecting the molecules based on their drug-like properties without any toxic moieties. The selected molecules that have an acceptable range of ADMET properties also possess a greater probability of being successful in clinical trials. Different ADMET parameters were obtained from the pkCSM software where only six compounds were selected based on their AMES and hepatotoxicity. The ADMET values of these six molecules are tabulated in Table 2. The molecules that show no toxicity qualify to be investigated further as AKT1 kinase inhibitors.

### 3.3. Screening for Biological Activity Prediction: PASS Analysis

The six selected compounds were subjected to PASS analysis for their biological properties. The PASS server (Way2drug) accepts the SMILES format for ligand files and displays results based on Pa and Pi values [57]. The activity predicted also served as an important criterion for selecting the compounds that possessed anticarcinogenic or antimutagenic properties. The six compounds showed acceptable results; however, the two compounds tuberosin and villosol exhibited the most promising outcomes from PASS analysis (Table 3). Both compounds possessed anticarcinogenic properties and, hence, were finalized for detailed interaction analysis.

### 3.4. Interaction Analysis

**Figure 1:**
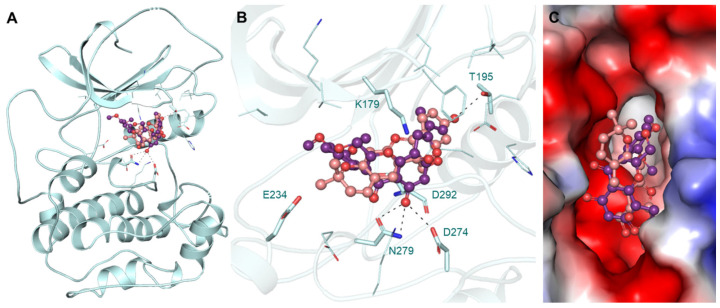
The schematic representation of the structure of AKT1 in a bound state with tuberosin and villosol. (**A**) Cartoon illustration of AKT1 with the elucidated molecules. (**B**) The representation of close interaction between AKT1 with tuberosin (red salmon) and villosol (purple). (**C**) The magnified illustration shows the surface potential view of the binding pocket of AKT1 with elucidated compounds.

The protein AKT1 and the selected compounds tuberosin and villosol were subjected to interaction analysis. The interaction data for the reference compound AZD5363 [63] were utilized for pose selection and the different conformations of docked tuberosin and villosol were superposed on the structure of AKT1. The results showed that tuberosin and villosol interacted with the crucial residues of the AKT1 that formed the binding pocket for ATP and known inhibitors (Figure 1A). The ATP binding site Lys179 and the active site residue Asp274 interacted with both compounds. The magnified view of the protein–ligand interactions is illustrated in Figure 1B. Following the known sites, tuberosin and villosol make various close interactions with the critical sites on AKT1. Both compounds block the binding site for ATP and the active site with virtuous complementarity (Figure 1C). These interaction outcomes indicate that the stability of both the compounds obstructs ATP accessibility to the protein and, therefore, they function as potent inhibitors of the protein.

**Figure 2:**
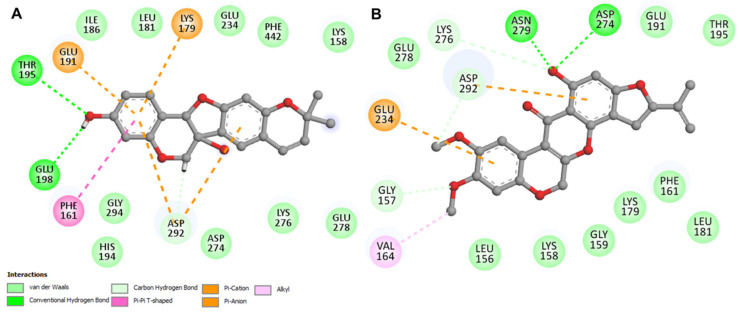
The two-dimensional representation of binding pocket residues of AKT1 and its respective interactions with compounds tuberosin (**A**) and villosol (**B**). The tuberosin shows the noncovalent interactions formed between the active site and ATP-binding residues of protein AKT1. The hydrogen bonds are formed between sites Lys179, Glu191, and Asp 292. The villosol shows noncovalent interactions between crucial residues of protein Glu234 and Asp292, with target protein AKT1.

The data from the interaction analysis is taken further for a detailed study against the target protein. The elaborate studies in the interaction unveil different types of noncovalent interactions that are formed between AKT1 and tuberosin or villosol. The two-dimensional plots of the probable interactions were obtained for both the compounds using Discovery Studio Visualizer. Both compounds interact with the Lys179 and Asp 274 residues on the ATP binding site and active site, respectively (Figure 2). Both compounds share common features of binding and, thus, can act as ATP-competitive inhibitors of AKT1. The selected conformations of both compounds were taken for all-atom MD simulations for 100 ns.

### 3.5. MD Simulations

The MD simulations provide insights into the flexibility of proteins with and without the presence of ligands [34,36,58,64]. Along with evaluating the interaction and flexibility of proteins and ligands, MD simulation fills in the structural details and dynamic behavior of the proteins which were not accomplished through experimental interventions [65]. The docked complexes with AKT1 were subjected to simulation in an explicit solvent environment to analyze their structural dynamic properties and stability index as a function of time for a time period of 100 ns.

#### 3.5.1. AKT1 Structural Changes through Ligand Binding

**Figure 3:**
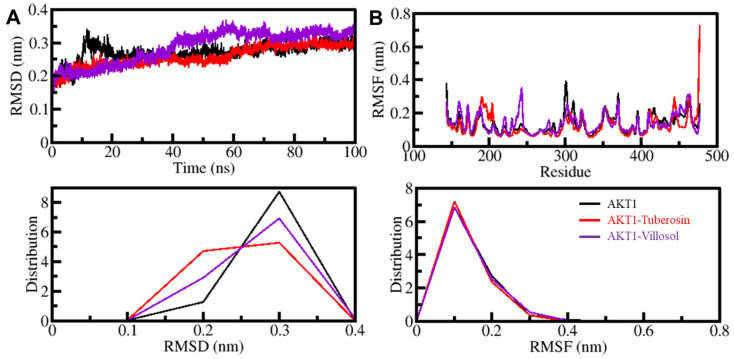
The structural dynamics of AKT1 after binding with tuberosin and villosol based on the function of time. (**A**) RMSD graph showing AKT1 when bound with tuberosin and villosol. (**B**) The average residual fluctuations (RMSF) graph of AKT1 is associated with tuberosin and villosol. The plots shown in the lower panels depict the probability distribution as PDF.

In order to assess the structural changes in the protein, the root-mean-square deviations (RMSDs) are crucial as they help to estimate the deviations in protein over time [35]. The alterations present in the backbone of the AKT1 were characterized by the RMSD analysis. The outcomes of RMSD analysis of the protein complexes AKT1-tuberosin and AKT1-villosol are represented in the form of time evolution plots (Figure 3). These plots show deviations occurring in the protein backbone and the results of these simulations are shown in Figure 3A. The data on simulation trajectory were utilized for analysis of the stability of the complexes. The data derived from the RMSD plot showed a fair consistency with small fluctuations. The deviations were reduced in the ligand–protein systems, especially in the AKT1-yuberosin complex. The three systems of apo-AKT1, AKT1-yuberosin, and AKT1-villosol reached equilibrium and endured stable conformation through the simulation trajectory of 100 ns (Figure 3A). The presence of random alterations in the AKT1-villosol complex’s RMSD of approximately 0.1 nanometers is distributed at 40–50 ns, which indicates a small adjustment in the system. The RMSD plots as a probability density function (PDF) depict lower RMSD and stabilization of AKT1 dynamics when the protein is present in the form of the complex. Consequently, the inference from the RMSD analysis is that the ligand-bound protein complex is folded and stable during the simulation.

The root-mean-square fluctuations (RMSFs) assist in the estimation of the residual vibrations present in the protein molecule at the time of the MD simulation [66,67]. The protein backbone was subjected to RMSF studies to evaluate the flexibility of the residues independently (Figure 3B). The figure depicts that the three systems show a similar pattern of RMSF and the residual fluctuations are minimized and stabilized when AKT1 is bound to tuberosin and villosol. Through RMSF intervention it was observed that residues in the protein binding pocket are almost stable and only show slight fluctuation at the time of the simulation. Only a few residues showed a deviation higher than the other systems, implying initial adjustment of the binding as seen in the RMSD analysis. Any major fluctuations in the AKT1 structure are only local to the loop regions and, comprehensively, tuberosin and villosol show stable interactions with AKT1.

**Figure 4:**
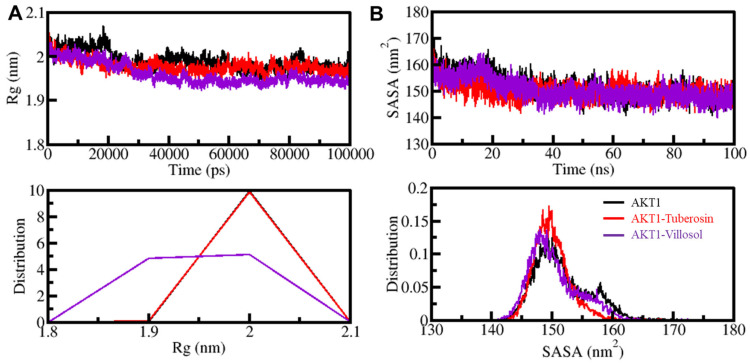
The structural compactness of AKT1 when present with tuberosin and villosol binding as a function of time. (**A**) The radius of gyration (*Rg*) through time evaluation. (**B**) SASA plot of AKT1 as a function of time before and after binding with selected compounds. Lower graph plots depict the probability density function (PDF).

The radius of gyration (*Rg*) is the parameter associated with the tertiary structure of a protein [68]. It indicates the root-mean-square distance of the collection of atoms from the collective center of mass. The parameter is extensively used as the means to evaluate the compactness seen in the tertiary structure of the protein. The determination of *Rg* is conducted on the basis of a time scale for apo and protein–ligand complex systems. After a rigorous assessment of the three systems, the plot indicates that the protein AKT1 in the presence of the compounds tuberosin and villosol shifted from the stability range of 2.05 nm to 1.90 nm during the simulation trajectory. The shift in the values is depicted in Figure 4. 

Following interaction analysis and estimation of *Rg*, the studies on the surface area of the protein and its accessibility to the adjacent solvent form an important part of the protein structure analysis [58]. The SASA, or solvent-accessible surface area, helps to study the stability of the protein structure and its folding patterns. Upon SASA analysis, we can understand the number of native contacts that are formed by the protein. The time-based evaluation of the SASA values against the protein AKT1 in its free and complex form were plotted (Figure 4B). The plot depicts no significant fluctuations in the SASA values during the entire simulation, even when bound to the two ligand molecules (Figure 4B). The overall compactness and the protein folding remain undisturbed as suggested by SASA analysis and, while correlating the values with *Rg*, the SASA distribution illustrates a similar equilibration pattern. The PDF distribution pattern of the AKT1 and its complex with tuberosin and villosol are shown in the lower panel of Figure 4B. 

#### 3.5.2. Dynamics of Hydrogen Bonds

**Figure 5:**
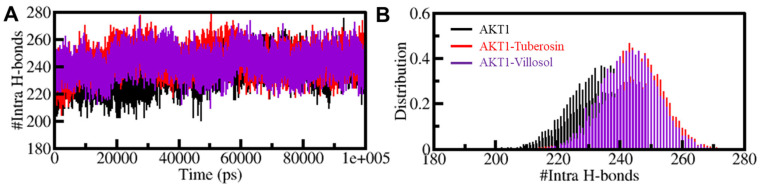
Formation of hydrogen bonds in time evolution plot formed within 0.35 nm of intra-AKT1 (**A**). The probability density function (PDF) of the intramolecular hydrogen bonds present within the structure of AKT1 (**B**).

Protein structures are dynamic in nature and are stabilized by the formation of crucial bonded interactions, i.e., intramolecular hydrogen bonds (H-bonds) and hydrophobic interactions [69,70]. The hydrogen bonds between the two participate in the folding of the protein and determining the stable protein conformation. The MD trajectories helped to estimate the formation of H-bonds in time evolution form. This analysis was essential in understanding the consistency of the intramolecular H-bonds in AKT1 when it is present in a bounded state. The changes in the number of H-bonds before and after binding with both compounds show that these bonds are more persistent and are significant in the geometry of the AKT1 structure. A slight increment in the number of intramolecular hydrogen bonds from the apostate to the bound state of protein AKT1 with tuberosin and villosol indicates that there is an increase in the compactness of the protein structure’s inbound state, thereby making it dynamically active. The PDF for the comparative change in the hydrogen bonds is depicted in Figure 5.

**Figure 6:**
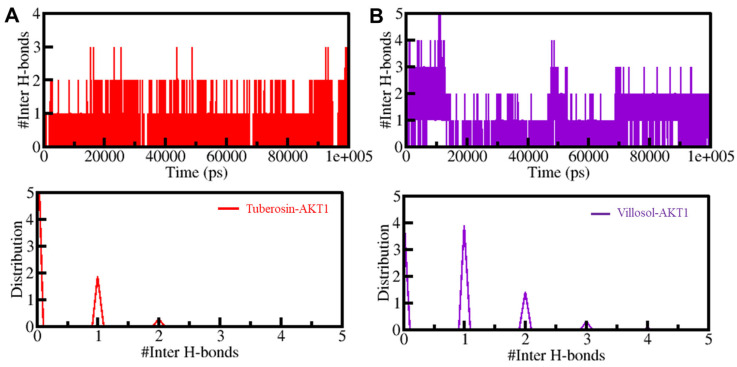
The time evolution of intermolecular hydrogen bonds formed within 0.35 nm between AKT1 and tuberosin (**A**) and villosol (**B**). The panels on the lower side depict the probability density function distribution of H-bonds.

The intramolecular H-bonds were studied through time evolution and the persistence of these bonds between the selected compounds and AKT1 was further investigated. The average number of H-bonds formed between the protein–ligand complexes is one (Figure 6). The presence of acceptable consistency in any system is shown by a higher PDF value and an average of one H-bond; such values are observed in AKT1-tuberosin and AKT1-villosol. There was no major change observed in the initial docking position of tuberosin and villosol, which proves the stability of both complexes. 

### 3.6. Principal Components Analysis 

**Figure 7:**
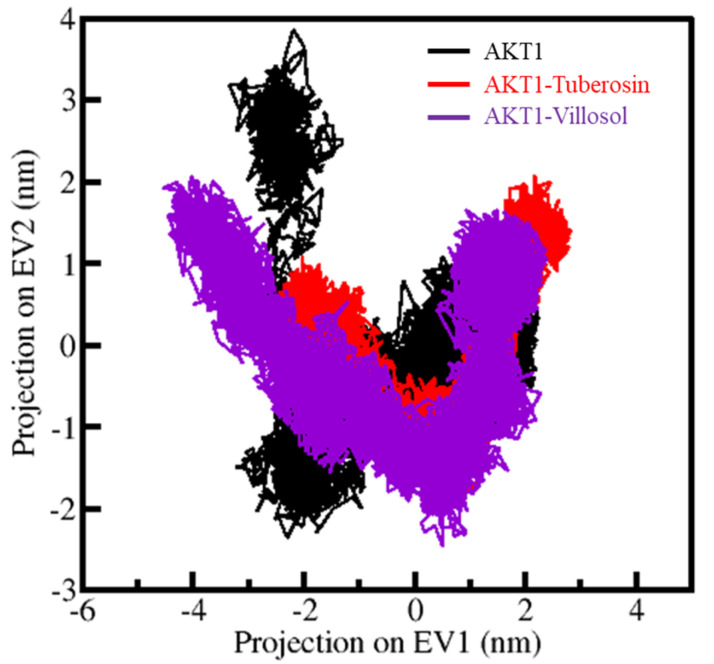
The conformational sampling in principal compound analysis (PCA). The two-dimensional projections illustrate the conformational sampling of AKT1-tuberosin and AKT1-villosol complexes.

The principal component analysis (PCA) provides information on the conformational sampling and the constitutive motions of the protein as seen in the simulated trajectories [71]. The PCA studies were performed on AKT1 and its complexes through the essential dynamics approach. The utilization of C^α^ atoms during the sampling resulted in generating the contour maps, as illustrated in Figure 7. The plot conveys that the AKT1-tuberosin and AKT1-villosol complexes are present in similar subspaces, as shown in both the EVs. The restricted movement in the essential subspace of AKT1-tuberosin with reduced flexibility can be seen on the EV2, which further supports the observations that the docked complexes are stable during the simulation studies (Figure 7).

### 3.7. Free Energy Landscapes

**Figure 8:**
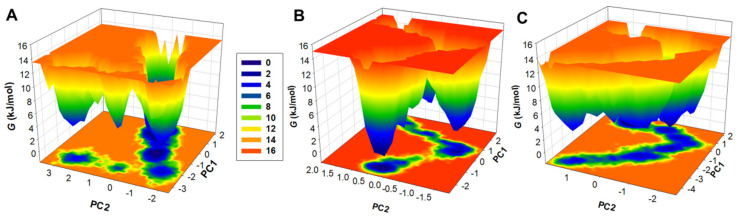
The free energy landscapes (FELs) of (**A**) AKT1, (**B**) AKT1-tuberosin complex, and (**C**) AKT1-villosol complex.

The free energy landscapes, or the FELs, are a vital means to explain the mechanism and patterns of protein folding [72]. The plots help to examine the levels of protein stability in the presence and absence of ligands, as well as under different solvent conditions during the MD simulation studies. The plots for the FELs for the three systems were generated and show the energy minima and conformational landscape in two principal components, i.e., PC1 and PC2. The plots are shown in the form of contoured maps represented in different colours, indicating the levels of the protein folding state. Figure 8 depicts that the binding of AKT1 to tuberosin and villosol causes fluctuations in the size and the positions of the stable global minima. The areas shown in dark blue denote the stable conformations with lower energy as the conformation is close to the native state of the protein (Figure 8). The unbound protein AKT1 is restricted to a large single global minima which expands to two or three basins. The AKT1-tuberosin and AKT1-villosol (Figure 8B–C) also show similar areas of global minima but with varying populations. Comprehensively, results obtained through the essential dynamics of AKT1 in free and bound states with tuberosin and villosol portray stability with minimal conformational switching during the simulations. 

## 4. Conclusions

Cancer has become one of the most challenging diseases, as it requires constant advancement in medical sciences and a better understanding of the underlying mechanisms of disease development and progression. Anticancer therapies are constantly evolving towards more efficient and targeted specific treatments. Various proteins and metabolic processes play essential roles in the development of cancer. AKT1 acts as a positive regulator of different types of cancers, especially OSCC. The pharmacological inhibition of AKT1 using tuberosin and villosol can be a novel strategy to develop more effective anticancer molecules. The computational analysis using state-of-the-art approaches has helped in identifying these two phytochemicals that have potential therapeutic features and show a significantly high affinity for AKT1. Tuberosin and villosol possess anticancer properties and further investigation of these compounds can contribute to the management of cancer. Thus, we present a proposition that tuberosin and villosol should be taken forward for further investigation in in vitro and in vivo settings for the development of better anticancer therapeutic regimes. This study also aimed to draw attention to the anticancer effects of traditional medicinal plants and their active components, promoting their use in developing potent anticancer therapies.

## Figures and Tables

**Table 1:** Top 30 hits against AKT1 and their docking scores.

S. No.	Compound ID	Phytochemical Name	Affinity (kcal/mol)	pKi	Ligand Efficiency
1.	174880	Lactupicrin	−10.1	7.41	0.34
2.	120698	Capnoidine	−10.0	7.33	0.37
3.	308140	Diospyrin	−10.0	7.33	0.36
4.	101277405	Arboreol	−10.0	7.33	0.36
5.	104940	Withanolide S	−9.9	7.26	0.39
6.	3822	Cubebol	−9.9	7.26	0.34
7.	5315739	N-Acetyldehydroanonaine	−9.8	7.19	0.43
8.	20055371	-	−9.7	7.11	0.40
9.	14630495	Tuberosin	−9.7	7.11	0.39
10.	146680	(2,2′-Binaphthalene)-5,5′,8,8′-tetrone, 1,1′-dihydroxy-6,6′-dimethyl-	−9.7	7.11	0.35
11.	16745513	4-Hydroxysesamin	−9.7	7.11	0.36
12.	5317297	5-(6,7-Dihydroxy-2-oxochromen-5-yl)-6,7-dihydroxychromen-2-one	−9.7	7.11	0.37
13.	101746	Sesamolin	−9.6	7.04	0.35
14.	12309624	Papaverrubine B	−9.6	7.04	0.34
15.	196979	Frangulin A	−9.6	7.04	0.32
16.	2913	Acora-4,10-diene	−9.5	6.97	0.43
17.	73393	Lagerine	−9.5	6.97	0.36
18.	21723446	Pseudostrychnine	−9.5	6.97	0.36
19.	5316097	Corylin	−9.5	6.97	0.39
20.	329584	3,3′-Bisjuglone	−9.5	6.97	0.36
21.	5281806	Psoralidin	−9.5	6.97	0.38
22.	119204	Roemerine	−9.4	6.89	0.45
23.	5320486	Gomezine	−9.4	6.89	0.47
24.	5459059	Ribasine	−9.4	6.89	0.36
25.	91572	Phaseolin	−9.4	6.89	0.39
26.	94577	Cepharadione A	−9.3	6.82	0.40
27.	101595	Vomicine	−9.3	6.82	0.33
28.	5320772	Psoralenol	−9.3	6.82	0.37
29.	5490819	Villosol	−9.3	6.82	0.31
30.	11438278	Cryptodorine	−9.2	6.75	0.40

**Table 2:** ADMET properties of the selected compounds.

S. No.	Compound ID	Phytochemical	Absorption	Distribution	Metabolism	Excretion	Toxicity
			GI Absorption (%)	BBB Permeation	*CYP2D6* Inhibitor	*OCT2* Substrate	*AMES* and Hepatotoxicity
1.	329584	3,3′-Bisjuglone	High	No	No	No	No
2.	5320772	Psoralenol	High	No	Yes	No	No
3.	5459059	Ribasine	High	Yes	Yes	No	No
4.	5490819	Villosol	High	No	Yes	No	No
5.	12309624	Papaverrubine B	High	Yes	Yes	No	No
6.	14630495	Tuberosin	High	Yes	Yes	No	No

**Table 3:** PASS analysis and molecular structures of the selected compounds.

S. No.	Phytochemical	Molecular Structure	Pa	Pi	Activity
1.	3,3′-Bisjuglone	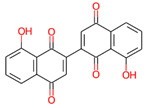	0866	0003	Antimutagenic
			0879	0016	Membrane integrity agonist
			0828	0009	Antineoplastic
			0799	0006	Kinase inhibitor
			0773	0007	Caspase-3 stimulant
2.	Psoralenol	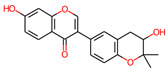	0704	0008	Anticarcinogenic
			0631	0023	Apoptosis agonist
			0535	0013	Antimutagenic
			0564	0053	Antineoplastic
			0506	0033	Kinase inhibitor
3.	Ribasine	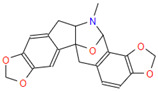	0878	0002	Neurotransmitter uptake inhibitor
			0512	0005	Antineoplastic alkaloid
			0474	0023	Caspase-8 stimulant
			0345	0079	Caspase-3 stimulant
			0276	0029	Antineoplastic (lymphoma)
4.	Villosol	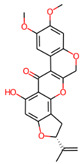	0993	0001	TP53 expression enhancer
			0902	0004	Apoptosis agonist
			0854	0007	Antineoplastic
			0662	0007	Antineoplastic (breast cancer)
			0556	0006	Prostate cancer treatment
5.	Papaverrubine B	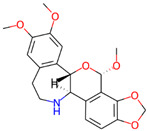	0683	0029	Antineoplastic
			0526	0028	Antineoplastic (non-Hodgkin lymphoma)
			0503	0006	Antineoplastic alkaloid
			0474	0009	Antimitotic
			0376	0027	Antineoplastic (lung cancer)
6.	Tuberosin	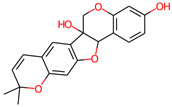	0792	0013	Antineoplastic
			0770	0005	Antineoplastic (breast cancer)
			0727	0021	TP53 expression enhancer
			0627	0023	Apoptosis agonist
			0571	0005	Antineoplastic (ovarian cancer)

## Data Availability

Not applicable.
